# Pulmonary function impairment of asymptomatic and persistently symptomatic patients 4 months after COVID-19 according to disease severity

**DOI:** 10.1007/s15010-021-01669-8

**Published:** 2021-07-28

**Authors:** Dieter Munker, Tobias Veit, Jürgen Barton, Pontus Mertsch, Carlo Mümmler, Andreas Osterman, Elham Khatamzas, Michaela Barnikel, Johannes C. Hellmuth, Maximilian Münchhoff, Julia Walter, Alessandro Ghiani, Stefan Munker, Julien Dinkel, Jürgen Behr, Nikolaus Kneidinger, Katrin Milger

**Affiliations:** 1 Department of Medicine V, University Hospital, LMU Munich, Marchioninistr. 15, 81377 Munich, Germany; 2 Comprehensive Pneumology Center Munich (CPC-M), Helmholtz Center and LMU Munich, Member of the German Center for Lung Research (DZL), Munich, Germany; 3 Max von Pettenkofer Institute and Gene Center, Virology, National Reference Center for Retroviruses, Ludwig Maximilian University (LMU) of Munich, Munich, Germany; 4 German Center for Infection Research (DZIF), Partner Site Munich, Munich, Germany; 5 Department of Medicine III, University Hospital, LMU Munich, Munich, Germany; 6 COVID-19 Registry of the LMU Munich (CORKUM), University Hospital, Ludwig-Maximilians University Munich, Munich, Germany; 7 Department of Pulmonology and Respiratory Medicine, Schillerhoehe Lung Clinic (affiliated to the Robert-Bosch-Hospital GmbH, Stuttgart), Solitudestrasse 18, 70839 Gerlingen, Germany; 8 Department of Medicine II, University Hospital, LMU Munich, Munich, Germany; 9 Department of Radiology, University Hospital, LMU Munich, Munich, Germany

**Keywords:** Post-COVID, Pulmonary function impairment, COVID-19, SARS-CoV-2

## Abstract

**Objective:**

Evaluation of pulmonary function impairment after COVID-19 in persistently symptomatic and asymptomatic patients of all disease severities and characterisation of risk factors.

**Methods:**

Patients with confirmed SARS-CoV-2 infection underwent prospective follow-up with pulmonary function testing and blood gas analysis during steady-state cycle exercise 4 months after acute illness. Pulmonary function impairment (PFI) was defined as reduction below 80% predicted of DLCOcSB, TLC, FVC, or FEV1. Clinical data were analyzed to identify risk factors for impaired pulmonary function.

**Results:**

76 patients were included, hereof 35 outpatients with mild disease and 41 patients hospitalized due to COVID-19. Sixteen patients had critical disease requiring mechanical ventilation, 25 patients had moderate–severe disease. After 4 months, 44 patients reported persisting respiratory symptoms. Significant PFI was prevalent in 40 patients (52.6%) occurring among all disease severities. The most common cause for PFI was reduced DLCOcSB (*n* = 39, 51.3%), followed by reduced TLC and FVC. The severity of PFI was significantly associated with mechanical ventilation (*p* < 0.001). Further risk factors for DLCO impairment were COPD (*p* < 0.001), SARS-CoV-2 antibody-Titer (*p* = 0.014) and in hospitalized patients CT score. A decrease of paO2 > 3 mmHg during cycle exercise occurred in 1/5 of patients after mild disease course.

**Conclusion:**

We characterized pulmonary function impairment in asymptomatic and persistently symptomatic patients of different severity groups of COVID-19 and identified further risk factors associated with persistently decreased pulmonary function. Remarkably, gas exchange abnormalities were revealed upon cycle exercise in some patients with mild disease courses and no preexisting pulmonary condition.

**Supplementary Information:**

The online version contains supplementary material available at 10.1007/s15010-021-01669-8.

## Introduction

The coronavirus disease 2019 (COVID-19) pandemic has put considerable strain on the health systems globally [1]. A significant percentage of patients develop COVID-19 pneumonia, which is the key determinant of prognosis in COVID-19 [2–4]. Risk factors for critical disease are mainly age, cardiovascular disease, diabetes and male sex [5]. Further pulmonary worsening is mediated by a cascade of hyperinflammatory responses, which is frequently observed after an initial period of symptom stability. COVID-19 pneumonia and the severe inflammatory process are hypothesized to cause lasting parenchymal damage. Besides, patients with moderate to severe COVID-19 disease are more likely to develop multi-organ disease, e.g. myocardial injury, thromboembolic complications, liver failure, and pneumothorax [6–10]. Comparably, patients after Acute Respiratory Distress Syndrome (ARDS) suffer from long-term physical problems and lung function deterioration [6].

An unknown proportion of patients may develop subclinical COVID-19 pneumonia with a mild disease course without hospitalization. So far, few studies have analyzed the impact of COVID-19 on lung function, and its long-term effects remain unclear. It is essential to identify risk factors for lasting pulmonary damage and define patients at risk who should receive specialized respiratory follow-up care after infection.

Here, we evaluate pulmonary function impairment after COVID-19 disease among all severity groups in persistently symptomatic and asymptomatic patients four months after infection. Furthermore, this study investigates predictors for lung function impairment according to disease severity in a highly diverse COVID-19 patient group, including ergometry assessment in a pulmonary aftercare setting.

## Methods

### Design and study population

This single-center cohort study included 76 patients with confirmed COVID-19 disease with acute illness during the first wave (March to August 2020). The Regional Ethics Committee approved the study of Ludwig-Maximilian-University (LMU) of Munich (project number 20-454), Germany. Up to December 2020, we prospectively included all adult patients (age > 18 years) who presented to the LMU outpatient department for follow-up 4 months after confirmed SARS-CoV-2 infection [positive polymerase chain reaction (PCR) or positive Anti-SARS-CoV-2 Immunoglobulin G (IgG) titer]. Hospitalized patients from LMU university hospital were referred to the outpatient clinic after discharge. Additionally, non-hospitalized (mild) cases were referred by the general practitioner. All patients provided written informed consent. Patients underwent a standardized interview to qualitatively evaluate COVID-19 related symptoms (yes or no) as previously described [11]. We differentiated between any COVID-19-related symptoms (including dyspnea, cough, fatigue, or anosmia, yes or no) and specific respiratory symptoms (dyspnea at rest or after exercise, cough, yes or no).

### Pulmonary function testing and cycle exercise blood gas analysis

Pulmonary function testing (PFT) was performed by plethysmography (Masterscreen Body, Jäger), and the following values were analyzed: Total lung capacity in liter (TLC in l and % predicted value), forced vital capacity (FVC in l and % predicted value), forced expiratory volume of 1 s (in l), Tiffeneau Index (FEV1/FVC), diffusion capacity of carbon monoxide by single breath test in mmol/minute/kiloPascal (DLCOcSB in mmol/min/kPa and % predicted value, corrected for hemoglobin). For plethysmographic ECSC [12] normal values were used, for spirometry GLI 2012 [13] normal values were used. For the definition of obstruction lower limit of normal (LLN) according to GLI 2012 was used (2).

If bronchial hyperresponsiveness was clinically suspected, inhaled methacholine testing was performed stepwise (Dosage: 0.05 mg, 0.15 mg, 0.45 mg). Bronchial hyperresponsiveness was diagnosed if FEV1 declined by > 20% at a methacholine concentration <  = 4 mg/ml (PC_20_ (provocative concentration causing a 20% fall in FEV1) [14].

Also, if bronchial obstruction (Tiffeneau Index < 70%) was detected, bronchodilator reversibility was tested using two puffs (100 mg/puff) of salbutamol [14].

Arterialized capillary blood gas analysis from the earlobe was performed at rest and after cycle ergometry. We preferred sampling from ear lobe capillaries in an outpatient setting due to the lower risk of complications including aneurysms, fistulas, ischemia infection, and hematoma. In the absence of contraindications, eligible patients performed steady-state cycle ergometry with stable resistance of 2 W/kg (ideal body weight) over 6 min with blood gas analysis performed during steady-state exercise at 5 min time point. Contraindications were severe resting hypoxia requiring long-term oxygen therapy, other medical conditions such as myocarditis, and incapacity to cycle due to orthopedic problems. As RQ was not directly measured, for calculation of AaDO2 under exercise, following simplified formula was used: AaDO2 = 140 (altitude Munich 530 m) – paO2 – paCO2.

### Diagnosis of COVID 19

We diagnosed COVID-19 in patients with matching symptoms and documented positivity for SARS-CoV-2 RNA in a reverse transcriptase-polymerase chain reaction (RT-PCR) from nasopharyngeal swabs (NPS) as described previously [15]. In case of high clinical suspicion but negativity for SARS-CoV-2 RNA in available clinical samples, e.g. false negatives due to limited quality of clinical sampling, absence of a PCR test due to low testing capacity at the beginning of the COVID-19 pandemic, we assumed a previous infection with SARS-CoV-2 after detection of specific antibodies against SARS-CoV-2.

### Anti-SARS-CoV-2 IgG detection

Serum samples were measured using Anti-SARS-CoV-2-IgG-ELISA (Euroimmun AG, Lübeck, Germany) at the time of presentation for PFTs. The assay was performed according to the manufacturer’s instructions. Borderline results were considered positive in this study.

### Disease severity and comorbidity

Patients were divided into 3 groups (mild, moderate/severe, and critical severity) according to WHO criteria (https://www.who.int/publications/i/item/clinical-management-of-covid-19). Moderate and severe disease courses were analysed as one group (moderate/severe). Symptomatic patients without hospitalization were classified as mild disease. Hospitalized patients with clinical signs of pneumonia without oxygen requirement were classified as moderate disease and patients who needed low-flow oxygen supplementation via nasal cannula were classified as severe disease. Critically ill patients received non-invasive ventilation (NIV) or invasive mechanical ventilation. Charlson Comorbidity Index was used to classify degree of comorbidity [16].

### Chest CT protocols and image analysis

If performed initially, CT scans were included in the analysis. All CT scans were performed at the end of inhalation using 64 row CT scanner with a detector configuration of 64 × 0.6 mm or using a 16-row CT scanner.

One radiologist and one pulmonologist screened the CT scans according to the radiologic scoring system from 0 to 25 points, which has been previously used to describe the extent of ground-glass opacities in SARS-CoV-2 infection [17–19].

### Nasopharyngeal swabs (NPS) and viral load analysis

NPS were routinely obtained on admission and according to local guidelines. NPS samples were taken on clinical suspicion of COVID-19. At admission, up to two NPS samples (with at least 12 h distance) and, if necessary, one sputum sample was obtained.

Repeated collection of either sample (NPS, sputum, and endotracheal aspirates) was performed in hospitalized patients for clinical monitoring purposes. When COVID-19 symptoms resolved and two consecutive NPS (at least with a day distance) showed a negative result, testing was stopped [15]. In this analysis, we used the term “duration of positivity for viral shedding.” It expresses the duration in days between the first and last confirmed positivity for SARS-CoV-2 in days. Different formulae were derived for each PCR assay to convert *Ct*/*Cp* values to copy number estimates as described previously [15, 20].

### Statistical analysis

Continuous variables were reported using mean with standard deviation (SD). We compared differences in means using unpaired *t*-test and ANOVA in the whole sample. When comparing repeated measurements of lung function testing, we used paired *t* test and Mann–Whitney test. Categorical variables were reported as absolute and relative frequencies and compared using *χ*^2^ test. In the univariate analysis we used Pearson correlation coefficient to test for association between variables. In the multivariate analysis, we applied multivariate linear regression to study the association of different covariates (e.g. SARS-CoV-2 IgG, Charlson Comorbidity Index, COPD, need for pulmonary ventilation) on changes in pulmonary function parameters (DLCOcSB, TLC, FVC, FEV1). Statistical significance was considered at *p* < 0.05. SPSS Version 26 and GraphPad Prism Version 9 were used for statistical analysis.

For descriptive purposes, pulmonary function impairment (PFI) was defined as a decrease of < 80% of predicted in any of the following: DLCOcSB, TLC, FVC, or FEV1.

## Results

### Baseline characteristics

**Fig. 1:**
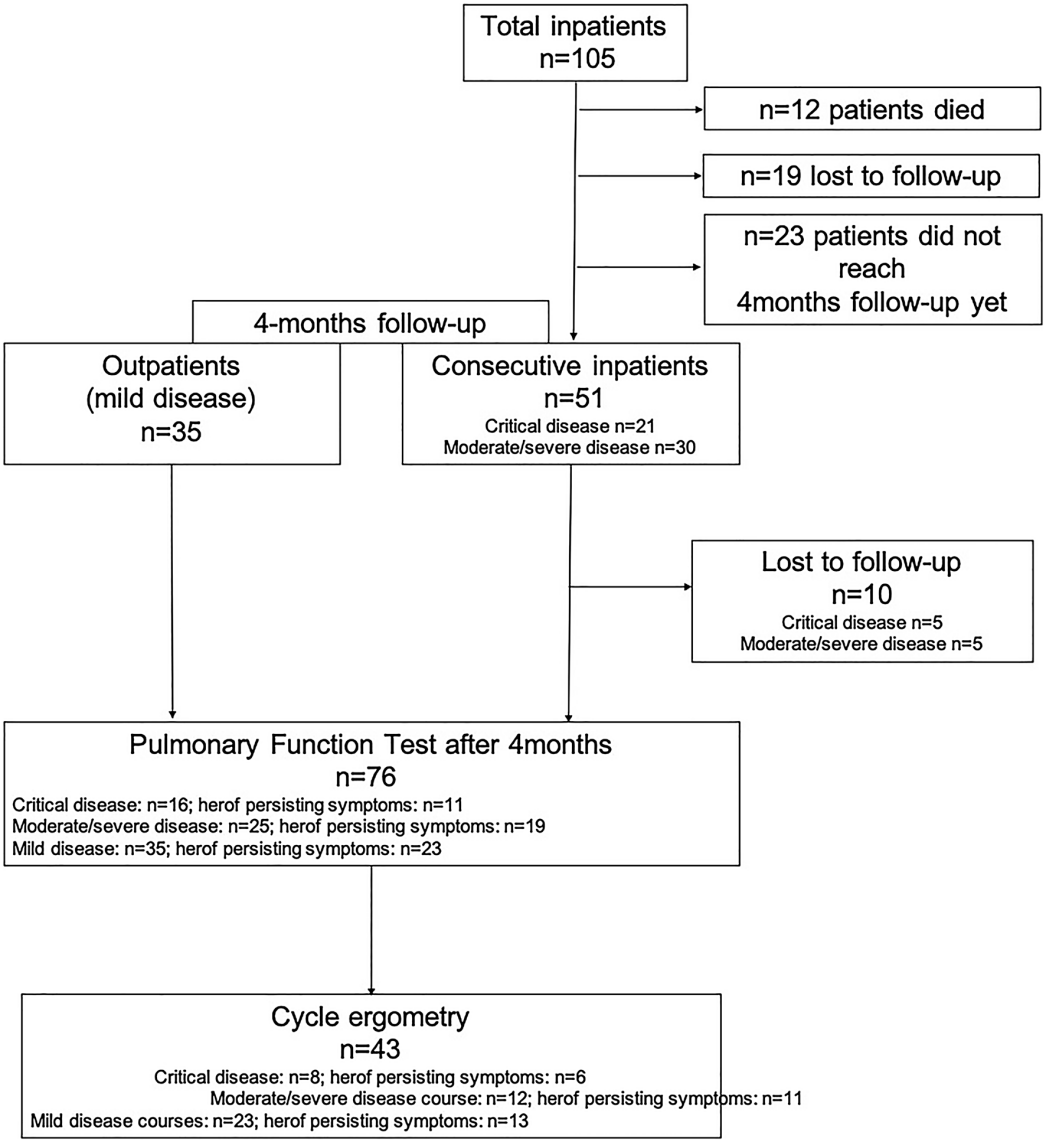
Study overview. Persisting symptoms: at least one symptom reported at 4 month-follow-up

Up to December 2020, 76 patients were included. The mean age (± SD) was 49.6 ± 17.4, and 43.3% of the patients were male. Thirty-five patients (46.1%) had mild disease treated as outpatients. In these mild patients, no imaging studies were performed during acute illness as patients were quarantined. 41 patients (53.9%) were hospitalized and had CT scan during acute illness. Hereof 25 patients (32.9%) had moderate/severe disease while 16 patients (21.1%) developed critical disease during hospitalization with the necessity for mechanical ventilation **(**Table 1). Patients with mild and moderate/severe disease were significantly younger than patients with critical disease with mean age (± SD) 44.3 ± 14.6, 48.1 ± 19.6 and 63.8 ± 10.3 years, respectively). 10 patients (24%) who were scheduled for appointments were lost to follow-up (Fig. 1).

**Table 1:** Baseline characteristics of the study population

	Total cohort	Mild No hospitalization	Moderate/severe disease Hospitalization, No mechanical ventilation	Critical disease Mechanical ventilation (non-invasive or intubation necessary)	*p* value
	*n* = 76	*n* = 35	*n* = 25	*n* = 16	
Age, mean ± SD	49.6 ± 17.4	44.3 ± 14.6	48.1 ± 19.6	63.8 ± 10.3	< 0.001*
Male, *n* (%)	33 (43.4)	13 (37.1)	9 (36.0)	12 (70.6)	0.285
Oxygen insufflation, *n* (%)	*n* = 28 (36.8)	n.a.	*n* = 12 (48.0%)	*n* = 16 (100.0%)	0.389
mechanical ventilation (days) ± SD	n.a.	n.a.	n.a.	19.1 ± 15.3	n.a.
Days of hospitalization ± SD	n.a.	n.a.	8.7 ± 5.9	39.2 ± 34.6	0.015*
Thoracic CT available in *n* (%) patients	45 (59.2%)	n.a.	19 (76%)	16 (100%)	0.489
CT score (range 0–25*)	n.a.	n.a.	5.8 ± 4.7	12.0 ± 7.4	< 0.001*
Peak viral load in nose swabs (copies/ml) ± SD	151.9 × 10^6^ ± 623.8 × 10^6^	n.a.	45.4 × 10^6^ ± 125.4 × 10^6^	310.7 ± 910.1 × 10^6^	0.337
Duration of viral shedding in days (last confirmed positivity) ± SD	17.6 ± 15.8	n.a.	16.0 ± 14.8	19.9 ± 17.3	0.131
Charlson Comorbidity Index ± SD, *n* (%)	1.3 ± 1.8	0.6 ± 1.3	1.7 ± 2.0	2.0 ± 1.9	0.007*
COPD, *n* (%)	5 (6.6%)	3 (8.6%)	1 (4.0%)	1 (6.3%)	0.136
Bronchial asthma, *n* (%)	9 (11.8%)	5 (14.3%)	2 (8.0%)	2 (12.5%)	0.073
History of smoking, *n* (%)	12 (15.8%)	5 (14.3%)	3 (12.0%)	4 (25.0%)	0.627
Arterial hypertension *n* (%)	17 (22.4%)	3 (8.6%)	7 (28.0%)	7 (43.8%)	0.071
Diabetes mellitus type 2, *n* (%)	4 (5.3%)	0 (0%)	2 (8.0%)	2 (12.5%)	0.553
Coronary artery disease, *n* (%)	5 (7.6%)	1 (2.9%)	2 (8.0%)	2 (12.5%)	0.462
Obesity, *n* (%)	8 (13.6%)	1 (2.9%)	3 (12.0%)	5 (31.3%)	0.045*
Obstructive sleep apnea, *n* (%)	1 (1.3%)	0	0	1 (6.3%)	0.39
Immunosuppression*, *n* (%)	9 (12.1%)	2 (5.7%)	3 (12.0%)	4 (25.0%)	0.232

Data are mean (SD) or *n* (%). *p* values were calculated by ANOVA, Mann–Whitney *U* test, or *χ*^2^ test, as appropriate. COVID-19 typical changes included either ground-glass opacities or diffuse bilateral infiltrates. Duration of nasopharyngeal viral shedding was defined by the time between symptom begin and last positivity for viral shedding in standardized nose swabs

^*^Defined as immunodeficiency due to primary or secondary hematologic disease *n* = 3 (3.9%) or current immunosuppressant medication due to rheumatic disease *n* = 6 (7.9%)

p-values<0.05 were considered significant and marked in bold

The majority of patients (*n* = 53, 69.7%) reported at least one persistent COVID-19 related symptom at presentation in our center four months after the initial diagnosis of COVID-19. Persistent respiratory symptoms (dyspnea at rest, dyspnea after exercise, or cough) were documented in 43 (56.6%) patients. 21 patients (27.6%) reported persistent fatigue, and 12 patients (15.8%) persistent anosmia (Table 2). One-third (24 patients) reported no residual symptoms at the follow-up presentation.

**Table 2:** Pulmonary function testing and blood gas analysis

	All patients	Mild disease Outpatients No hospitalization	Moderate/severe disease Hospitalization, No mechanical ventilation	Severe disease Mechanical ventilation (non-invasive or intubation necessary)	*p* value
Patients, *n*	*n* = 76	*n* = 35	*n* = 25	*n* = 16	
Persistent COVID-19-related symptoms*	53 (69.7%)	23 (65.7%)	19 (76.0%)	11 (68.8%)	0.435
Persistent COVID-19-related respiratory symptoms**	43 (56.6%)	15 (42.9%)	17 (68.0%)	11 (68.8%)	0.233
Spirometry					
TLC, L	5.8 ± 1.5	6.1 ± 1.3	5.9 ± 1.6	5.1 ± 1.7	0.11
TLC, % of predicted	96.3 ± 19.1	99.8 ± 11.4	101.8 ± 21.4	77.5 ± 19.8	< 0.01*
FVC, L	3.7 ± 1.1	4.0 ± 1.1	3.7 ± 0.8	2.9 ± 1.1	< 0.01*
FVC, % of predicted	97.1 ± 17.6	99.4 ± 13.9	102.5 ± 16.1	79.7 ± 20.0	< 0.01*
FEV1, L	3.0 ± 0.9	3.3 ± 0.9	3.1 ± 0.8	2.4 ± 0.9	< 0.01*
FEV1, % of predicted	97.0 ± 18.5	97.1 ± 14.5	104.3 ± 16.5	84.1 ± 23.5	< 0.01*
FEV1/FVC	83.5 ± 7.5	82.4 ± 7.3	84.6 ± 4.8	84.5 ± 23.5	0.46
Blood gases and diffusion					
DLCOcSB (mmol/min/kPa)	7.2 ± 2.2	7.9 ± 2.2	7.4 ± 1.5	5.5 ± 2.2	< 0.01*
DLCOcSB, % of predicted	77.2 ± 16.5	79.9 ± 15.5	81.4 ± 11.9	61.2 ± 18.0	< 0.01*
At rest					
paO2 (mmHg)	75.5 ± 9.2	78.7 ± 6.6	76.2 ± 8.0	67.3 ± 11.0	< 0.01*
paCO2 (mmHg)	36.2 ± 3.6	36.2 ± 3.6	35.4 ± 3.2	37.3 ± 4.2	0.94

Data are mean (SD) or *n* (%). *p *values were calculated by Mann–Whitney *U* test or *χ*^2^ test, as appropriate

*TLC* total lung capacity, *FVC* forced vital capacity, *FEV1* forced expiratory volume in one second, *Tiffeneau Index (FEV1/FVC) DLCOcSB* diffusing capacity of the lung for carbon monoxide single-breath corrected for hemoglobin, *pO2* capillary partial oxygen pressure, *pCO2* capillary partial carbon dioxide pressure

*Dyspnea, cough, fatigue, or anosmia

p-values<0.05 were considered significant and marked in bold

Comorbidities were frequent, with an average Charlson comorbidity score of 1.3 ± 1.8, with arterial hypertension (22.4%) and obesity (17%) being most common. Fourteen patients (18.4%) had a previously known chronic lung disease (COPD *n* = 5, 6.6%; bronchial asthma *n* = 9, 11.8%, Table 1). According to patient interview, no patient had been diagnosed with interstitial lung disease previously. Nine patients were immunocompromised (*n* = 3 with hematologic disorders, *n* = 6 under immunosuppressants due to rheumatic disease).

### Pulmonary function testing at 4-month follow-up

**Fig. 2:**
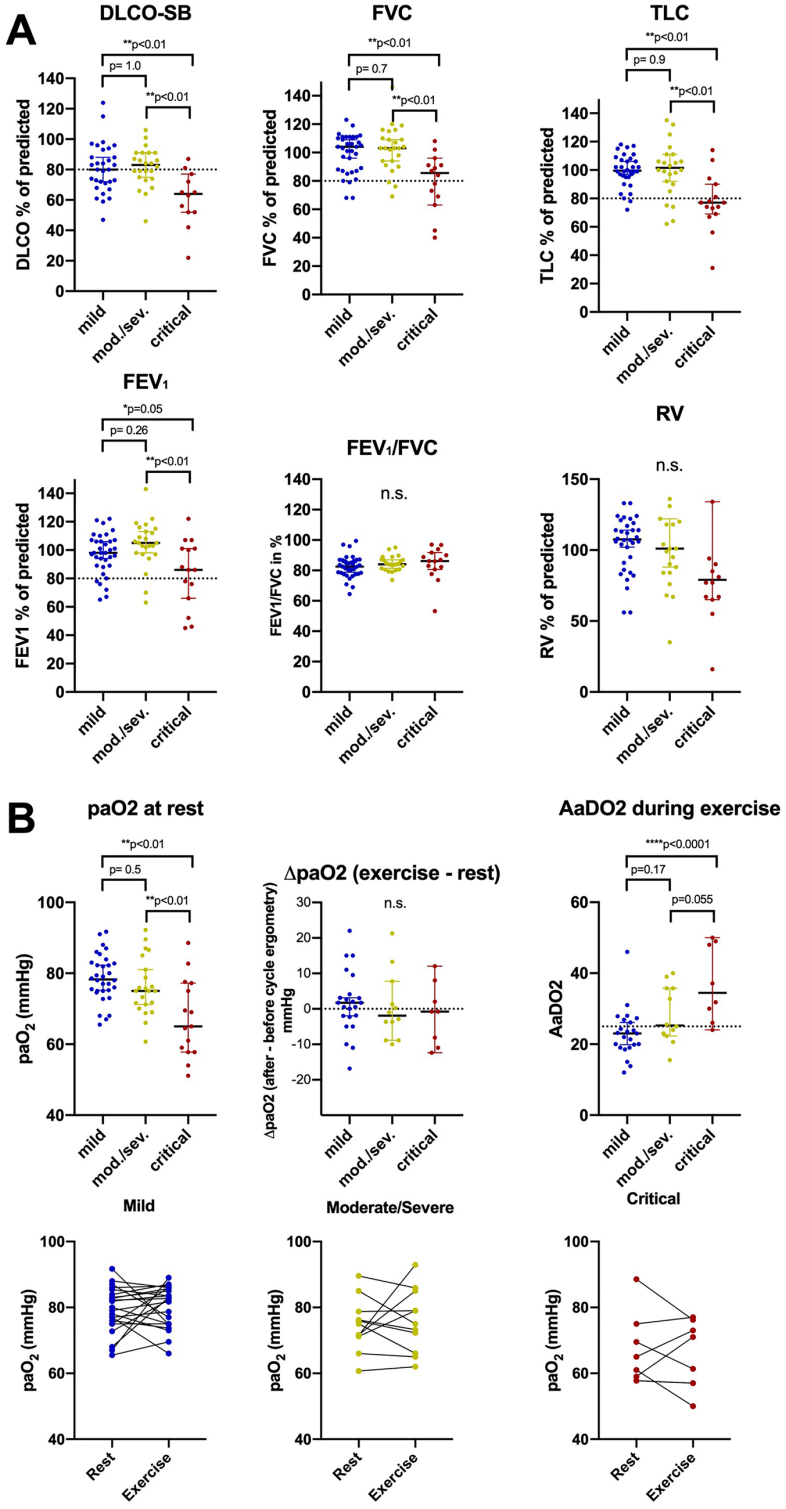
**A** Pulmonary function testing 4 months after acute illness in patients with mild, moderate/severe and critical disease course. Mean and individual values are displayed, all parameters are shown as % predicted. DLCOcSB diffusion capacity for CO Single breath, *FVC* Forced vital capacity, *TLC* Total lung capacity, *FEV1* Forced Expiratory Volume in 1 s, *RV* Residual Volume. Statistical testing performed by ANOVA + Tukey’s multiple comparison test. **B** paO2 and AaDO2 from arterialized capillary blood gas analysis at rest and during at exercise according to COVID-19 disease severity 4 months after acute illness

DLCOcSB was significantly lower after critical COVID-19 disease (60.6 ± 19.5% of predicted) compared to moderate/severe (81.4 ± 11.9% of predicted) and mild disease (79.9 ± 15.5% of predicted) courses (*p* < 0.01, Table 2 and Fig. 2A). Likewise, TLC, FVC, and FEV1 were significantly decreased after critical disease course, while mean FEV1/FVC was normal in all disease severities (Table 2 and Fig. 2A). Resting arterialized capillary partial oxygen pressure (paO2) was significantly lower (65.3 ± 10.2 mmHg) in patients after critical disease (*p* < 0.01) compared to moderate/severe (76.2 ± 8.0 mmHg) and mild disease (78.7 ± 6.6 mmHg) (Table 2 and Fig. 2B). Mean paCO2 was within normal range (36.2 ± 3.6 mmHg) and did not differ between the severity groups (*p* = 0.37). During cycle ergometry, blood gas analysis (BGA) was performed in 43 eligible patients (56.6%). During ergometry, paO2 decreased > 3 mmHg in 13 patients (30%). A paO2 decrease was noted in 3 out of 8 patients (38%) after critical COVID 19 disease, in 5 out of 12 patients (42%) moderate/severe disease, and 5 out of 23 patients (22%) with mild disease (Fig. 2B). AaDO2 under exercise was significantly higher in critical patients compared to the other groups, but using a cutoff of 25 mmHg abnormal values were also observed in patients after mild disease (Fig. 2B).

If FEV1/FVC indicated obstructive ventilatory defect (< LLN), bronchodilator reversibility with salbutamol was tested (*n* = 10). Reversibility was confirmed in one (10%) patient. Additionally, 8 patients with normal lung function but persisting cough or dyspnea were challenged with inhaled methacholine. Bronchial hyperresponsiveness was newly diagnosed in 3 patients after SARS-CoV-2 infection.

### Risk factors for pulmonary function impairment

Next, we investigated risk factors for pulmonary function impairment using univariate regression analysis. Here, mechanical ventilation, duration of mechanical ventilation, duration of hospitalization, oxygen insufflation, Charlson Index, COPD, smoking history, obesity and SARS-CoV2-IgG antibody titer were significantly correlated with pulmonary function parameters (Table 3a).

**Table 3:** (A) Univariate regression analysis; (B) multivariate regression analysis in the total cohort, (C) multivariate regression analysis in the hospitalized cohort (D) multivariate regression analysis in the non-critical cohort

A								
All patients, *n* = 76, univariate regression	DLCOcSB		TLC		FVC		FEV1	
	ULR		ULR		ULR		ULR	
	*p* value	Coeff. r	*p* value	Coeff. r	*p* value	Coeff. r	*p* value	Coeff. r
Mechanical ventilation (yes = 1, no = 0)	< 0.001**	**− 0.453**	< 0.001**	**− 0.488**	< 0.001**	**− 0.465**	0.002*	**− 0.349**
Duration of mechanical ventilation (days)	0.002**	**− 0.378**	< 0.001**	**− 0.401**	0.001*	**− 0.39**	0.020*	**− 0.27**
Duration of hospitalization (days)	0.002**	**− 0.378**	0.025*	**− 0.266**	**0.015**	**− 0.287**	0.141	− 0.175
Oxygen insufflation necessary (yes = 1, no = 0)	0.006**	**0.332**	0.003**	**− 0.342**	0.019*	**− 0.727**	0.312	− 0.119
Charlson Index	0.001*	**− 0.401**	0.088	− 0.201	0.085	− 0.201	0.121	− 0.182
COPD (yes = 1, no = 0)	0.010*	**− 0.313**	0.006**	**− 0.319**	0.007*	**− 0.312**	0.002*	**− 0.355**
	0.191	− 0.162	0.158	− 0.167	0.372	− 0.116	0.106	− 0.189
Bronchial asthma (yes=1, no=0)								
Pack years	0.005*	**− 0.339**	0.097	− 0.196	0.006*	**− 0.319**	0.001*	**− 0.376**
Type 2 diabetes (yes = 1, no = 0)	0.802	0.031	0.905	0.014	0.756	− 0.037	0.703	0.045
Coronary artery disease (yes = 1, no = 0)	0.244	− 0.144	0.414	− 0.097	0.11	− 0.188	0.223	− 0.143
Obesity (yes = 1, no = 0)	0.042*	**− 0.25**	0.003**	**− 0.348**	< 0.001**	**− 0.44**	0.001*	**− 0.374**
Immunosuppression (yes = 1, no = 0)	0.09	− 0.209	0.742	0.039	0.979	0.003	0.392	− 0.101
SARS-CoV-2 IgG Antibody titer	0.003**	**− 0.385**	0.001**	**− 0.398**	0.033*	**− 0.269**	0.331	− 0.115
Persistent symptoms	0.454	0.092	0.45	− 0.088	0.214	− 0.144	0.295	− 0.12
Persistent respiratory symptoms	0.076	0.205	0.937	− 0.009	0.438	− 0.09	0.985	0.002

B								
All patients, *n* = 76, multivariate regression	DLCOcSB		TLC		FVC		FEV1	
	MLR		MLR		MLR		MLR	
	*p* value	Coeff. beta	*p* value	Coeff. beta	*p* value	Coeff. beta	*p* value	Coeff. beta
Mechanical ventilation (yes = 1, no = 0)	0.009**	**− 0.308**	< 0.001*	**− 0.49**	< 0.001**	**− 0.506**	0.001**	**− 0.352**
Duration of mech. ventilation	0.913	0.016	0.608	− 0.08	0.781	− 0.043	0.894	− 0.022
Duration of hospitalization (days)	0.51	0.086	0.472	− 0.099	0.427	0.102	0.546	0.089
Oxygen insufflation necessary (yes = 1, no = 0)	0.44	0.113	0.732	0.048	0.589	0.07	0.146	0.216
Charlson Index	0.282	0.126	0.059	0.198	0.065	− 0.547	0.256	− 0.128
COPD (yes = 1, no = 0)	0.007**	**− 0.264**	0.001**	**− 0.318**	0.003**	**− 0.296**	0.004**	**− 0.312**
SARS-CoV-2 IgG Antibodies	0.014*	**− 0.291**	0.105	− 0.199	0.245	− 0.134	0.845	− 0.026

C								
Hospitalized, *n* = 41, multivariate regression	DLCOcSB		TLC		FVC		FEV1	
	MLR		MLR		MLR		MLR	
	*p* value	Coeff. beta	*p* value	Coeff. beta	*p* value	Coeff. beta	*p* value	Coeff. beta
Computertomography Score (0–25)	**0.026**	**− 0.323**	0.006*	**− 0.419**	0.006**	**− 0.419**	0.66	− 0.071
Mechanical ventilation (yes = 1, no = 0)	0.003**	**− 0.425**	0.033*	**− 0.321**	0.033*	**− 0.321**	0.001**	**− 0.514**
Duration of intubation in days	0.431	− 0.145	0.083	− 0.245	0.083	− 0.245	0.9	− 0.025
Oxygen insufflation necessary (yes = 1, no = 0)	0.166	− 0.213	0.126	− 0.216	0.506	− 0.102	0.867	0.028
SARS-CoV-2 IgG Antibodies	0.042*	**− 0.3**	0.313	− 0.153	0.761	− 0.047	0.94	− 12

D								
Mild versus moderate/severe *N* = 60	DLCOcSB		TLC		FVC		FEV1	
	MLR		MLR		MLR		MLR	
	*p* value	Coeff. beta	*p* value	Coeff. beta	*p* value	Coeff. beta	*p* value	Coeff. beta
Duration of hospitalization in days	0.857	− 0.022	0.059	0.241	0.067	0.231	0.819	− 0.028
Oxygen insufflation necessary (yes = 1, no = 0)	0.651	− 0.054	0.818	0.03	0.591	0.068	0.599	− 0.064
Charlson Index	0.771	0.041	0.026*	− 0.287	0.254	− 0.16	0.225	0.179
COPD (yes = 1, no = 0)	0.039*	**− 0.251**	0.407	− 0.116	0.03	− 0.281	0.047	− 0.233
Immunocompromised (yes = 1, no = 0)	0.002**	**− 0.391**	0.69	0.053	0.286	− 0.136	< 0.001*	− 0.476
SARS-CoV-2 IgG Titer	0.050*	**− 0.237**	0.72	− 0.047	0.974	0.004	0.039*	− 0.249

p-values<0.05 were considered significant and marked in bold

Using multivariate regression analysis (MLR), the strongest risk factor for persistent pulmonary function impairment in the total cohort (*n* = 76) was mechanical ventilation (DLCOcSB: *p* = 0.009*, TLC: *p* < 0.001*, FVC: *p* < 0.001, FEV1: *p* = 0.001; Table 3b). Further, COPD was associated with reduced DLCO, TLC, FVC, FEV1 after correcting for influences by other variables in multivariate regression analysis and SARS-CoV-2 IgG titer at 4 months was negatively correlated with DLCO among all subgroups (MLR: *p* = 0.014, *p* = 0.042, Table 3b). Looking at the inpatients (*n* = 41) with CT scans available during hospital stay, MLR revealed additional negative correlations between DLCO at 4 months and CT scores during hospital stay (*p* = 0.026) (Table 3c). In non-critical patients (mild + moderate/severe: *n* = 60) there was no correlation of oxygen insufflation and length of hospitalization to pulmonary function parameters, whereas preexisting immunosuppression was associated with reduced DLCO (Table 3d).

### Pulmonary function impairment (PFI) in persistently symptomatic versus non-symptomatic post-COVID-19 patients

Significant pulmonary function impairment (PFI) defined as any value < 80% predicted, was detected in 40 (52.6%) patients**.** As there was a trend for an association of respiratory symptoms and reduced pulmonary function parameters in univariate regression analysis (Table 3a), we further investigated a possible association of symptoms and PFI. No significant difference appeared in lung function parameters when comparing asymptomatic and patients with persisting COVID-19-related symptoms (Figure S1). Likewise, looking only at patients with mild disease courses, symptomatic and asymptomatic patients had no significant differences in lung function parameters (DLCO: *p* = 0.267; TLC: *p* = 0.814, FVC: *p* = 0.823, FEV1: *p* = 0.991), and proportions of patients with impaired pulmonary function were similar among persistently symptomatic and asymptomatic patients (Table S1). Within the mild disease group, persistently symptomatic (all COVID-19-related symptoms) patients displayed hyperventilation compared to asymptomatic patients (*n* = 35, paCO2: 35.0 ± 2.9 vs. 38.3 ± 6.5 mmHg, *p* = 0.019; paO2: 76.4 ± 6.9 vs 75.5 ± 11.1, *p* = 0.862).

A drop of paO2 > 3 mmHg during exercise occurred in 15% asymptomatic (*n* = 2 of 13) and in 37% (*n* = 11 of 30, Figure S1) of symptomatic (all COVID-19 related symptoms) patients.

## Discussion

Here we report comprehensive pulmonary function evaluation of COVID-19 patients 4 months after acute illness in a broad cohort of patients ranging from mild to critical disease course including patients with persisting symptoms as well as patients who were asymptomatic at the time-point of follow--up.

We found impaired pulmonary function in more than half of the patients with a reduction in DLCO as the most frequent impairment (95% of patients with impairment). Recent studies reported reduced DLCO, TLC, and FVC one to six months after COVID-19 disease in selected patients; however, so far, most studies focused on hospitalized cohorts [21–26].

Contrary to these studies, we included 46% (*n* = 35) patients who had a mild disease course without hospitalization and also 33% (*n* = 25) who reported no persistent COVID-19-related symptoms. Even though patients with critical disease had more severe PFI at mean, we also found PFI defined as values < 80% predicted in a proportion of patients who had mild and moderate/severe COVID-19.

Reduced DLCO and lung capacities after COVID-19 may indicate interstitial lung damage. Interstitial lung disease after COVID-19 disease is an important clinical manifestation in the convalescent phase. Recent studies report up to 62% of pulmonary interstitial fibrosis-like changes two months after critical disease courses [27, 28]. Similar to ARDS, long-term mechanical ventilation likely plays an important role in the development of interstitial fibrosis. Even though we found mechanical ventilation was a main risk factor for DLCO impairment in this study, a significant percentage of moderate to severe patients without the need for mechanical ventilation had decreased DLCO (40%). As preexisting COPD was rare, this suggests non-critical COVID-19 pneumonia as likely cause in most patients. Additionally, impaired perfusion could play a role, as thromboembolism and alveolar capillary microthrombi have been described in COVID-19 [29].

Long-term follow-up has not been established yet. It is unclear whether impaired diffusion capacity will improve and which risk factors for persistence over more extended time courses exist. Interestingly in SARS, DLCOi (< 80%) was only observed in 12.7% of patients at 3 months after acute illness [30]. Early identification of restrictive disease and identification of risk factors might be essential for early treatment interventions, and primarily measurement of DLCO appears crucial in patients after COVID-19. TLC and FVC are less frequently impaired and thus secondary markers for identifying patients at risk. If progressive fibrosing interstitial lung disease after COVID-19 is confirmed by further diagnostic assessment, antifibrotic agents, e.g. Nintedanib might be a treatment option [31], that needs to be studied in RCTs.

To elucidate risk factors for pulmonary function impairment, we integrated clinical data during acute illness. As mentioned above, the most substantial risk factor for impaired DLCO, TLC, FVC, and FEV1 was mechanical ventilation, in line with findings from other recent cohorts [21, 22, 24, 32]. Interestingly, elevated SARS-CoV-2 Titer independently predicted impaired DLCO among all patients. Higher levels of antibody response have been described in critical versus non-critical COVID-19 disease [33]. Deeper analyses found that 2 weeks after presentation IgG antibodies of patients with critical disease compared to those with moderate disease showed functional characteristics likely to induce monocyte/macrophage activation [34]. Thus antibody responses have been suggested to play a role in an exaggerated inflammatory response and immunopathology [35], with possibly detrimental effects on lung tissue. These mechanisms might explain consecutive diffusion impairment in our study.

Including only the inpatients with CT scan available during acute illness (*n* = 41), an additional negative predictor for DLCO impairment was increased initial CT score (MLR: *p* = 0.049).

To further investigate gas exchange after COVID-19, we implemented steady-state cycle ergometry with blood gas analysis in eligible patients. Of note, a decrease of capillary paO_2_ > 3 mmHg or increased AaDO2 > 25 mmHg after cycle ergometry occurred after mild disease courses (8 of 23, 35% and 7 of 23, 30%), revealing gas exchange abnormalities even in some patients who did not require hospitalization. Rinaldo et al. [36] recently performed cardiopulmonary exercise testing (CPET) with maximal exercise in 75 COVID-19 survivors three months after discharge. The researchers mainly reported impaired exercise capacity probably caused by muscular deconditioning in 41 patients (54.7%) compared to 34 patients with normal exercise capacity while mean AaDO2 was 26 in both groups.

Also, among mild and moderate/severe patients (*n* = 60), pulmonary function impairment did not depend on the duration of hospitalization (*p* = 0.857) and oxygen insufflation (*p* = 0.651). These findings might indicate the presence of undiagnosed viral pneumonia and subsequent interstitial lung damage or perfusion abnormalities after mild disease courses.

Furthermore, our post-COVID-19 patients underwent a detailed pulmonologic evaluation. Postviral bronchial hyperreactivity syndrome is common after respiratory tract viral infections [37]; however, its prevalence after COVID-19 is unclear. Here, we investigated bronchial hyperresponsiveness only in patients with normal baseline lung function but persisting respiratory symptoms. We report 3 out of 8 challenged patients who had bronchial hyperresponsiveness after COVID-19, suggesting this may also be a complication of COVID-19. In total, only in 3.9% patients bronchial hyperresponsiveness was confirmed, indicating a minor role of prior postulated postviral bronchial hyperreactivity [37, 38]. The main symptoms were shortness of breath and persistent cough. All patients with bronchial hyperresponsiveness were treated with Beclometasone/Formoterol 100 µg/6 µg 2–0–2 for 6–8 weeks and symptoms resolved in all patients within three months.

As expected, COPD was associated with impaired pulmonary function in this cohort. While these patients have preexisting reduced pulmonary function due to COPD, it is also a known risk factor for all-cause mortality and progression to severe disease in COVID-19 patients [39].

Moreover, smoking and pack-years are negatively correlated with lung function decline in UR. Pathophysiological mechanisms of smoking may lead to declined lung function before infection or contribute to worsening lung condition due to higher vulnerability.

At 4 months after acute illness, persistent COVID-19 specific symptoms occurred in all subgroups (*n* = 52; 68.4%), but patients with mild disease course were more frequently free of symptoms than the other groups. Interestingly, we found no significant correlation between persisting symptoms and impaired pulmonary function, indicating that other mechanisms may play an important role in the reported symptoms. Diverse mechanisms for long-/post-COVID symptoms have been proposed, but the exact pathophysiology remains elusive so far [40].

Contrary to other studies, we have also included patients with mild disease course and patients who did not report symptoms at follow-up and thus analyzed the influence of disease severity and persistent symptoms on PFI. This approach revealed that PFI might also be present after mild COVID-19, and such patients should also be included in future studies.

However, it should be noted that this was not a population-based study. At the time of presentation in our aftercare unit 23 patients (65.7%) had persistent symptoms, whereas 12 patients (34.3%) negated persistent COVID-19 related symptoms. Asymptomatic patients (*n* = 12) were referred or presented themselves due to intrinsic or scientific interest after announcing the post-COVID-19 aftercare offer after the first COVID-19 wave. Nevertheless, we assume that negative effects on lung function are less frequent in patients who do not present in an aftercare unit after mild disease courses. Potential selection bias might be present in the mild disease course group. Thus, the frequencies of symptomatic versus asymptomatic follow-up patients are not representative for an overall post-COVID-population. Additionally, analyses are limited due to moderate sample size, and symptoms were analyzed only qualitatively. As PFT values before the disease were not available for the majority of the patients, mild preexisting abnormalities cannot be excluded. Furthermore, in this study, we performed steady-state exercise and not complete cardiopulmonary exercise testing (CPET). Interestingly, in mild disease, paCO2 was at mean 3.3 mmHg lower in symptomatic patients than in asymptomatic patients, but clinical significance of this small difference is currently unclear.

According to our data and recent publications, we recommend pulmonary aftercare to patients after severe and critical COVID-19 disease courses [11, 23, 32, 39, 41]. In particular, patients with initial elevated CT values and after mechanical ventilation should be considered for referral. In addition, patients with preexisting lung conditions (COPD), former or active smokers, and patients with persistent pulmonary symptoms (cough, dyspnea, fatigue) might profit from pulmonary assessment. If after diagnostic evaluation (lung function, CT scan, echocardiography and others) persistent symptoms remain unclear, CPET might be an option to discriminate deconditioning from severe other causes [36].

In sum, we describe that PFI may be present in symptomatic and asymptomatic post-COVID-19 patients but is most frequent in those who had severe acute illness. Interestingly, a decrease of paO2 upon exercise was found in 1/5 of the mild disease patients, most of whom had borderline-mild DLCO impairment at rest. Thus, exercise testing should be included in post-COVID-19 evaluation as it may reveal subtle gas exchange abnormalities, which could be responsible for some of the persisting symptoms.

## Supplementary Information

Below is the link to the electronic supplementary material.

Supplementary file2 Figure S1: Pulmonary function testing and arterialized capillary blood gas analysis at rest and during exercise in asymptomatic patient and patients with persisting symptoms at 4months after acute illness. Mean and individual values are displayed, all parameters are shown as % predicted. DLCOcSB diffusion capacity for CO Single breath, FVC = Forced vital capacity; TLC = Total lung capacity, FEV1 Forced Expiratory Volume in 1sec. RV = Residual Volume. Statistical testing performed by t-test (JPG 469 kb)

Supplementary file2 (DOCX 38 kb)
