# Supplementary figures and images for: Pulmonary function impairment of asymptomatic and persistently symptomatic patients 4 months after COVID-19 according to disease severity
Source: Infection. 2021 Jul 28;50(1):157–68. doi: 10.1007/s15010-021-01669-8 (PMC8318328; doi:10.1007/s15010-021-01669-8)

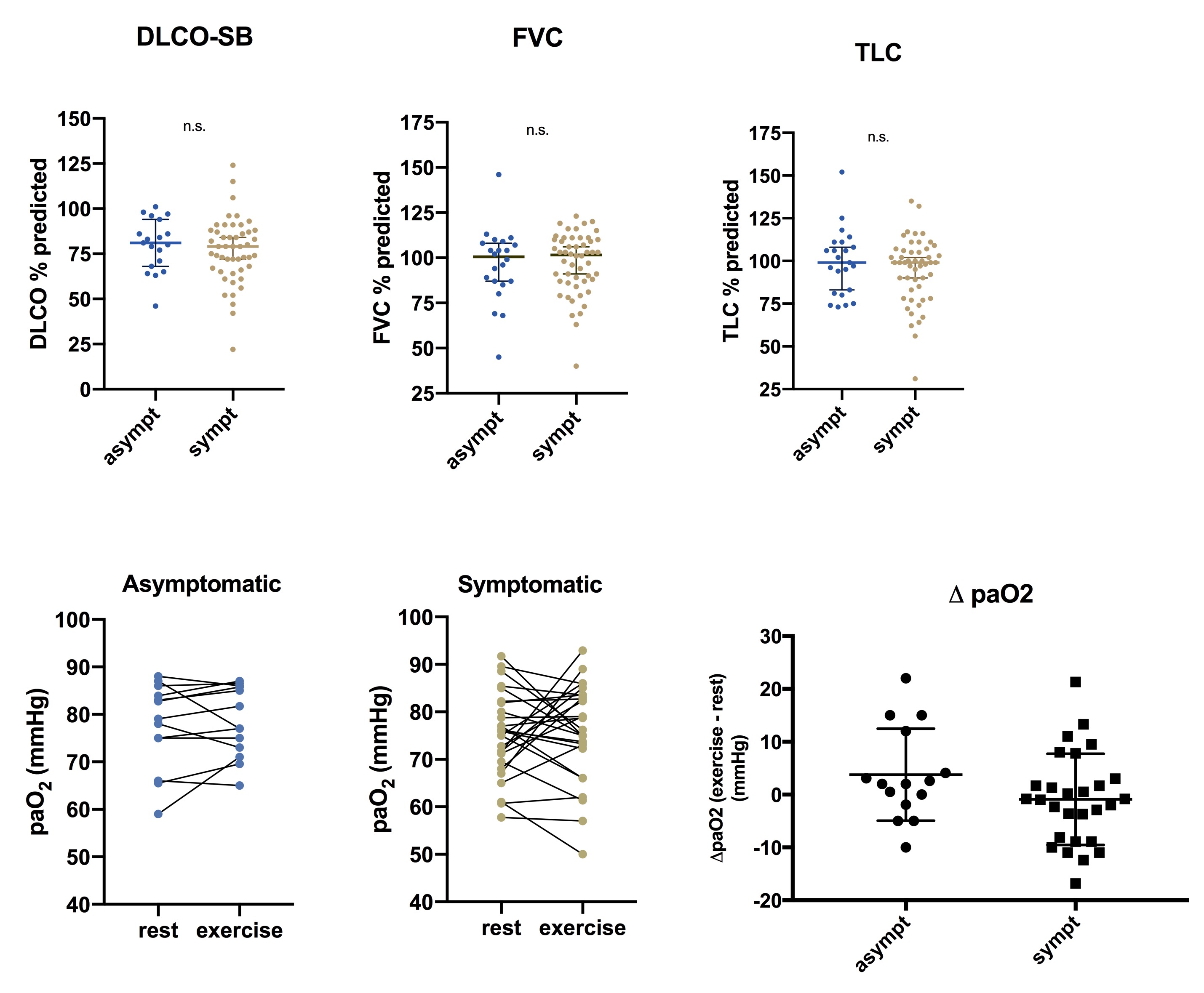

Supplement: Supplementary file 1 — Supplementary file2 Figure S1: Pulmonary function testing and arterialized capillary blood gas analysis at rest and during exercise in asymptomatic patient and patients with persisting symptoms at 4months after acute illness. Mean and individual values are displayed, all parameters are shown as % predicted. DLCOcSB diffusion capacity for CO Single breath, FVC = Forced vital capacity; TLC = Total lung capacity, FEV1 Forced Expiratory Volume in 1sec. RV = Residual Volume. Statistical testing performed by t-test (JPG 469 kb) [file 15010_2021_1669_MOESM1_ESM.jpg]
