# Supplementary material for: Pulmonary function impairment of asymptomatic and persistently symptomatic patients 4 months after COVID-19 according to disease severity
Source: Infection. 2021 Jul 28;50(1):157–68. doi: 10.1007/s15010-021-01669-8 (PMC8318328; doi:10.1007/s15010-021-01669-8)
Supplement: Supplementary file 2 — Supplementary file2 (DOCX 38 kb) [file 15010_2021_1669_MOESM2_ESM.docx]

|  | **COVID-19** | **related symptoms** | **No reported** | **persistent symptoms** | **p-value** |
| --- | --- | --- | --- | --- | --- |
| **Critical disease**  Inpatients, mech. Ventilation | PFi: | 11 of 11 (100%) | PFi: | n=5 of 5 (100%) | 1 |
|  | DLCOcSBi: | n=9 of 11 (81.8%) | DLCOcSBi: | n=4 of 5 (80.0%) | 0.931 |
|  | FVCi: | n=5 of 11 (45.5%) | FVCi: | n=2 of 5 (40.0%) | 0.839 |
|  | TLCi: | n=8 of 11 (72.7%) | TLCi: | n=2 of 5 (40.0%) | 0.210 |
|  | FEV1i: | n=4 of 11 (36.4%) | FEV1i: | n=2 of 5 (40.0%) | 0.889 |
|  | HAR: | n=9 of 11 (81.8%) | HAR: | 2 of 5 (40.0%) | 0.094 |
|  | EID: | n=3 of 6 (50%) | EID: | n= 0 of 2 (0.0%) | 0.338 |
| **Moderate/Severe course**  Inpatients, no mech. Ventilation | PFi: | n=7 of 19 (36%) | PFi: | n=3 of 6 (50%) | 0.566 |
|  | DLCOcSBi: | n=8 of 19 (42.1%) | DLCOcSBi: | n=3 of 6 (50%) | 0.734 |
|  | FVCi: | n=1 of 19 (5.3%) | FVCi: | n= 2 of 6 (33%) | 0.065 |
|  | TLCi: | n=2 of 19 (10.5%) | TLCi: | n=2 of 6 (33%) | 0.184 |
|  | FEV1i: | n=1 of 19 (5.3%) | FEV1i: | n=1 of 6 (16.7%) | 0.369 |
|  | HAR: | n=4 of 19 (21.1%) | HAR: | n=1 of 6 (16.7%) | 0.815 |
|  | EID: | n=5 of 11 (45.5%) | EID: | n=0 of 1 (0%) | 0.506 |
| **Mild disease course**  Outpatients | PFi: | n=11 of 23 (47%) | PFi: | n=6 of 12 (50%) | 0.903 |
|  | DLCOcSBi: | n=10 of 23 (43.5%) | DLCOcSBi: | n=5 of 12 (41.7%) | 0.918 |
|  | FVCi: | n=2 of 23 (8.7%) | FVCi: | n= 1 of 12 (8.3%) | 0.971 |
|  | TLCi: | n=2 of 23 (8.7%) | TLCi: | n=0 of 12 (0%) | 0.293 |
|  | FEV1i: | n=4 of 23 (17.4%) | FEV1i: | n=1 of 12 (8.3%) | 0.467 |
|  | HAR: | n=3 of 23 (13.0%) | HAR: | n=1 of 12 (8.3%) | 0.678 |
|  | EID: | n=3 of 13 (23.1%) | EID: | n=2 of 10 (20%) | 0.887 |

**Table S1.** Distribution of symptomatic and asymptomatic patients according to pulmonary function impairment (PFi) and disease courses. PFi: defined by <80% of predicted of DLCOcSB, FVC, TLC or FEV1.

DLCOi, FVCi, TLCi and FEV1i (impairment) respectively defined by capacity <80% of predicted. HAR: Hypoxemia at rest. EID: Exercise-induced decrease of paO2 >3mmHg.

|  |  | **Below 80% of predicted** | **Below Lower Limit of Normal**  **(LLN)** | **p-value** |
| --- | --- | --- | --- | --- |
| **Critical disease**  Inpatients, mech. Ventilation  **n=16** | PF: | n= 16 (100%) | n= 13 (81.3%) | 0.225 |
|  | DLCOcSB: | n=15 (93.8%) | n=9 (56.3%) | 0.014 |
|  | FVC: | n=7 (43.8%) | n=5 (31.3%) | 0.465 |
|  | TLC: | n=10 (62.5%) | n=7 (43.8%) | 0.288 |
|  | FEV1: | n=6 (37.5%) | n=4 (25.0%) | 0.446 |
| **Moderate/Severe course**  Inpatients, no mech. Ventilation  **n=25** | PF: | n=10 (40%) | n=9 (36.0%) | 0.771 |
|  | DLCOcSB: | n=11 (44.0%) | n=6 (24.0%) | 0.232 |
|  | FVC: | n=3 (12.0%) | n=1 (4.0%) | 0.272 |
|  | TLC: | n=4 (16.0%) | n=3 (12.0%) | 0.684 |
|  | FEV1: | n=2 (8.0%) | n=2 (8.0%) | 1.0 |
| **Mild disease course**  Outpatients  **n=35** | PF: | n=17 (48.6%) | n=14 (40.0%) | 0.470 |
|  | DLCOcSB: | n=15 (42.9%) | n= 13 (37.1%) | 0.626 |
|  | FVC: | n=3 (8.7%) | n=2 (5.7%) | 0.971 |
|  | TLC: | n=2 (5.7%) | n=1 (2.9%) | 0.643 |
|  | FEV1: | n=5 (14.3%) | n=3 (8.6%) | 0.452 |

**Table S2. Comparison of predefined lung function impairment by <80% of predicted (PFi, DLCOi, FVCi, TLCi and FEV1i) and below lower limit of normal (LLN).** P-values were calculated by χ² test and considered significant <0.05.
